# Comparison of the interactions of daunorubicin in a free form and attached to single-walled carbon nanotubes with model lipid membranes

**DOI:** 10.3762/bjnano.7.46

**Published:** 2016-04-08

**Authors:** Dorota Matyszewska

**Affiliations:** 1 Faculty of Chemistry, Biological and Chemical Research Centre, University of Warsaw, Żwirki i Wigury 101, 02089 Warsaw, Poland

**Keywords:** daunorubicin (DNR), 1,2-dipalmitoyl-*sn*-glycero-3-phosphothioethanol (DPPTE), drug carriers, model lipid membranes, single-walled carbon nanotubes (SWCNTs)

## Abstract

In this work the interactions of an anticancer drug daunorubicin (DNR) with model thiolipid layers composed of 1,2-dipalmitoyl-*sn*-glycero-3-phosphothioethanol (DPPTE) were investigated using Langmuir technique. The results obtained for a free drug were compared with the results recorded for DNR attached to SWCNTs as potential drug carrier. Langmuir studies of mixed DPPTE–SWCNTs-DNR monolayers showed that even at the highest investigated content of the nanotubes in the monolayer, the changes in the properties of DPPTE model membranes were not as significant as in case of the incorporation of a free drug, which resulted in a significant increase in the area per molecule and fluidization of the thiolipid layer. The presence of SWCNTs-DNR in the DPPTE monolayer at the air–water interface did not change the organization of the lipid molecules to such extent as the free drug, which may be explained by different types of interactions playing crucial role in these two types of systems. In the case of the interactions of free DNR the electrostatic attraction between positively charged drug and negatively charged DPPTE monolayer play the most important role, while in the case of SWCNTs-DNR adducts the hydrophobic interactions between nanotubes and acyl chains of the lipid seem to be prevailing. Electrochemical studies performed for supported model membranes containing the drug delivered in the two investigated forms revealed that the surface concentration of the drug-nanotube adduct in supported monolayers is comparable to the reported surface concentration of the free DNR incorporated into DPPTE monolayers on gold electrodes. Therefore, it may be concluded that the application of carbon nanotubes as potential DNR carrier allows for the incorporation of comparable amount of the drug into model membranes with simultaneous decrease in the negative changes in the membrane structure and organization, which is an important aspect in terms of side effects of the drug.

## Introduction

**Figure 1:**
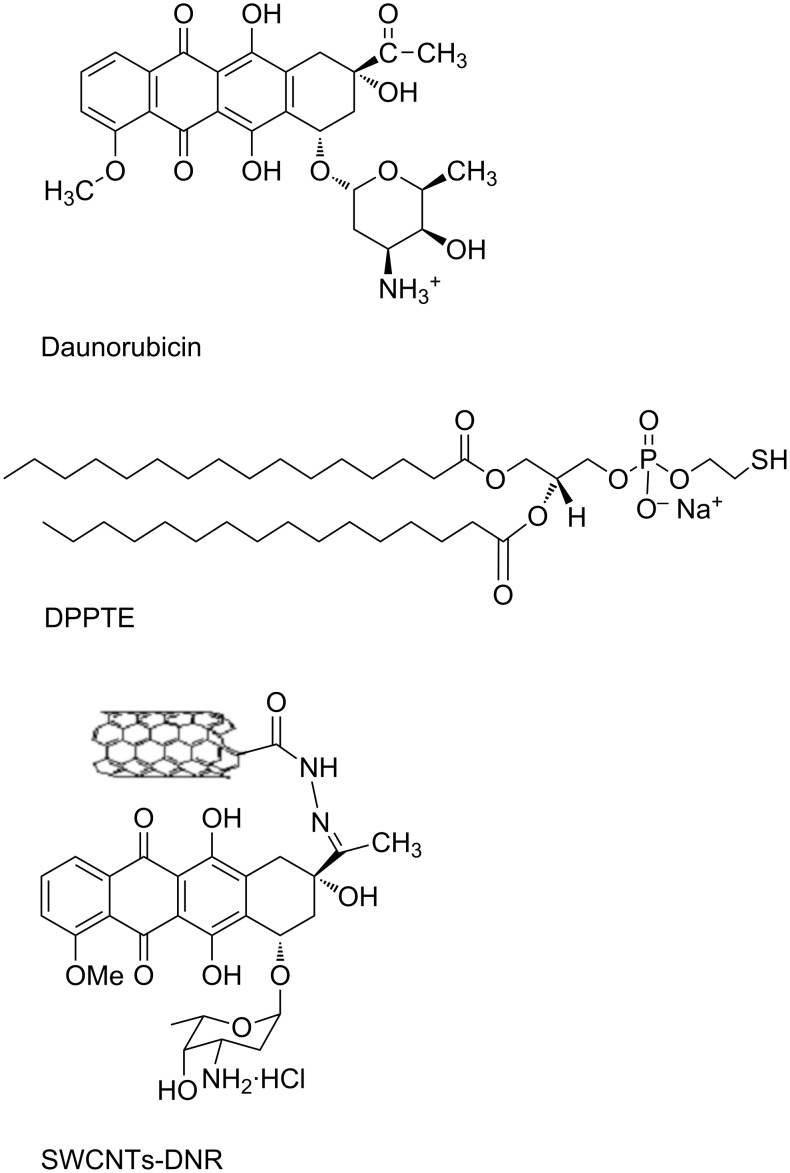
Structures of daunorubicin (DNR) and 1,2-dipalmitoyl-*sn*-glycero-3-phosphothioethanol (DPPTE) used as a component of model membranes and single-walled carbon nanotubes modified with daunorubicin (SWCNTs-DNR).

Daunorubicin (DNR) is an anthracycline antitumor drug, which finds application in the treatment of various types of cancer including leukemia, breast cancer, ovarian cancer, lung carcinoma and several sarcomas (Figure 1). Its mode of action consists in the intercalation into DNA double strand, which leads to the inhibition of the process of duplication and transcription of mRNA as well as DNA damage by the inhibition of topoisomerase II [1–2]. However, the second mechanism involving the increased production of ceramides inside cells has been recently postulated [3]. Application of this drug in the cancer treatment is limited because of serious side effects including drug-induced heart failure, which is mainly associated with the process of the reactive oxygen species formation as well as the formation of hydroxyl radicals by free iron cations in the Fenton reaction [4]. Therefore, numerous studies focus on the application of different drug delivery systems (DDS) to transport daunorubicin to cancer cells [5].

Drug delivery systems are aimed at providing enhanced transport of therapeutic agents directly to the targeted organs and tissues, which enables the elimination or significant decrease in the side effects of a drug. One of the most common type of drug nanocarriers includes liposomes, which are commercially available daunorubicin formulation (Daunoxome^®^) used in the treatment of Kaposi’s sarcoma [6]. Despite that, there are still numerous studies on the improvement of this drug delivery system aiming at enhancing drug loading into cells by using specific interactions between targeting agents and their receptors, such as for example folates and transferrin [7–8]. Additionally, liposomes are also prepared in such a way that simultaneous loading of two drugs into a liposome in order to improve the efficiency of the treatment is possible [9]. Dual drug loading is also employed in case of other DDS such as biodegradable polymers, which co-assemble into composite micelles [10]. Another type of common drug carriers includes nanoparticles. Magnetic Fe_3_O_4_ nanoparticles are often employed because they give possibility to control the transport by applying external magnetic fields. Such magnetic nanoparticles conjugated with DNR were reported to induce apoptosis of cancer cell lines [11–12]. Other examples of nanoparticles include titanium dioxide (TiO_2_) and gold nanoparticles (AuNPs) [13–14]. In the latter case the nanoparticles were also modified with aptamer – single-stranded DNA or RNA sequences showing high specificity and affinity to their targets, which were employed as molecular targeting agents for targeted drug transport.

Carbon nanotubes (CNTs) are among the promising drug delivery systems. They attract scientists’ attention due to their properties such as stability, robustness, high drug carrying capacity and ability to penetrate cell membranes [15]. Although toxicity of the nanotubes is an issue, it strongly depends on the dimensions and type of functionalization, which may significantly increase their biocompatibility [16–17]. There are different mechanisms proposed to explain the cellular uptake of CNTs including the passive diffusion in a non-invasive manner (tiny nanoneedle mechanism) [18]. Carbon nanotubes have been successfully used to transport different types of anticancer agents including camptothecin, doxorubicin and daunorubicin [19]. The two main methods of attaching the drugs comprise either covalent attachment or physical adsorption based on π–π stacking interactions. There are a few reports in the literature on the preparation and characterization of CNTs-DNR adducts used as drug delivery systems. In those works daunorubicin was conjugated to either polyethylene glycol (PEG) functionalized single-walled carbon nanotubes (SWCNTs) [20] or to aptamer-wrapped SWCNTs via π–π interactions. In both cases the cytotoxicity of the conjugates was verified on the selected cancer cell lines.

In this study the influence of both free daunorubicin and daunorubicin attached via covalent bond to single-walled carbon nanotubes (SWCNTs, Figure 1) on model biological membranes was investigated using Langmuir technique. The model membranes were composed of 1,2-dipalmitoyl-*sn*-glycero-3-phosphothioethanol (DPPTE, Figure 1). This thiolipid has been used so far either as a tethering layer in tBLM systems employed, e.g., for studying ion-channel proteins on electrode surfaces or as a model membrane in the studies of a lipolytic enzyme, phospholipase A2, which was employed as a tool for modifying the structure of supported thiolipid layers in a controlled way [21–22]. Due to the presence of a thiol group in the polar headgroup region, this lipid has negative charge and it is possible to transfer monolayers onto solid support by means of both Langmuir–Blodgett method and self-assembly, which results in the differences in the packing of the thiolipid molecules in the supported layers depending on the mode of transfer [23]. Our previous results of Langmuir studies show that daunorubicin in a free form may easily incorporate into the DPPTE layers during their formation and significantly change the properties of the layers [24]. The effect of daunorubicin attached to carbon nanotubes as potential drug carrier on the properties of model membranes is compared to that reported for a free drug. In order to obtain more detailed information on the interactions between DNR in the two investigated forms the electrochemical techniques were also employed to study the supported lipid layers containing both free DNR and SWCNTs-DNR adducts.

## Experimental

### Langmuir monolayers at the air–water interface

Langmuir monolayers were prepared using a KSV LB trough 5000 (KSV Ltd., Finland) equipped with hydrophilic barriers and a Wilhelmy balance made of a filter paper used as a surface pressure sensor. The experiment was controlled with software version KSV 5000. 1,2-dipalmitoyl-*sn*-glycero-3-phosphothioethanol (sodium salt, DPPTE, Avanti Polar Lipids) was dissolved in chloroform to give 1 mg/mL stock solution. Carbon nanotubes used for the modification were commercial, oxidized nanotubes SWCNT(s)-COOH (CheapTubes, Brattleboro, USA) with the diameter of 1–2 nm and length of 5–30 µm. The modification at the ends of the nanotubes as well as in the defect sites was obtained by the formation of SWCNTs-end hydrazide, which was then mixed with daunorubicin to form hydrazone. The detailed procedure of the covalent end modification of single-walled carbon nanotubes with daunorubicin by the formation of hydrazone was inspired by the protocol previously described for side and end carboxylated SWCNTs modification [25]. Basing on the TGA analysis such modification procedure yields the functionalization degree in the order of approximately 1.25 × 10^−7^ mol of drug per 1 mg of carbon nanotubes (approximately 1.5 × 10^−3^ mol of drug per 1 mol of carbon). Mixed DPPTE–SWCNTs-DNR dispersion was prepared by weighing approximately 0.5 mg of modified nanotubes and adding the appropriate volume of DPPTE stock solution in order to obtain the desired weight ratio of carbon nanotubes to thiolipid. Prior to the deposition at the air–water interface, the mixed solutions were sonicated in the ultrasonic bath (Emag, Germany) for approximately 30 min to ensure carbon nanotubes dispersion. Langmuir monolayers were prepared on either Milli-Q ultra-pure water (resistivity 18.2 MΩ/cm) subphase (mixed DPPTE–SWCNTs-DNR monolayers) or subphase containing daunorubicin (AK Scientific, USA). After careful cleaning of the subphase, a few drops of the solution (DPPTE or mixed DPPTE–SWCNTs) were spread on the subphase using a Hamilton microsyringe and the solution was left for approximately 15 min for solvent evaporation. Barrier speed during compression was 10 mm/min (7.5 cm^2^/min). Experiments were performed at room temperature (21 ± 1 °C).

### Langmuir–Blodgett transfer

Supported DPPTE or mixed DPPTE–SWCNTs-DNR monolayers were prepared by means of Langmuir–Blodgett method. Prior to the transfer, gold electrodes (10 × 10 mm slides, Ssens, The Netherlands), which were 200 nm thick gold films evaporated onto borosilicate glass precoated with an underlayer of chromium, were flame annealed, cleaned in the mixture of H_2_O_2_/NH_3_/H_2_O with 1:1:5 ratio at 70 °C for approximately 5 min and thoroughly rinsed with Milli-Q water. The DPPTE and mixed DPPTE–SWCNTs-DNR layers were deposited on the gold surface by vertical withdrawal of the electrode at the speed of 25 mm/min to give a transfer ratio of 1.0 ± 0.1. The target surface pressure, at which the layers were transferred was equal to 30 mN/m and 35 mN/m for DPPTE and mixed DPPTE–SWCNTs-DNR monolayers, respectively. After the LB deposition and before the electrochemical measurements, the electrodes were dried in air for approximately 2 h.

### Electrochemical experiments

Electrochemical experiments were performed using AutoLab AUT 71819 with the GPES 4.9 software in the three electrode system with Ag/AgCl as a reference electrode and platinum foil (10 × 10 mm plate) as a counter electrode. Phosphate buffer (50 mM, pH 6.9) prepared from sodium phosphates (Avantor, Poland) was used as a supporting electrolyte.

## Results and Discussion

### Monolayer studies at the air–water interface

**Figure 2:**
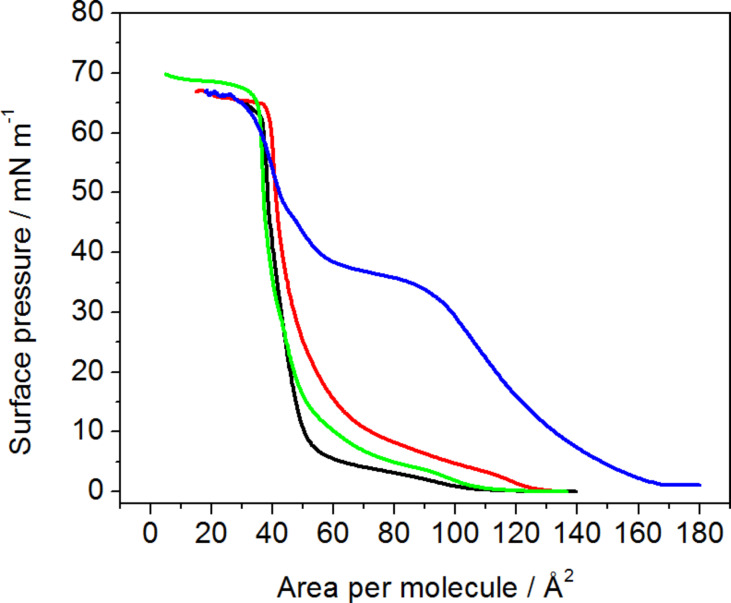
Surface pressure-area per molecule (π–A) isotherms of DPPTE monolayer on water subphase (black), mixed layers of DPPTE:SWCNTs-DNR weight ratio of 1:5 (red), mixed layers of DPPTE/SWCNTs-DNR weight ratio of 10:1 (green), DPPTE monolayer on subphase containing 10^−5^ DNR (blue).

In order to study the influence of daunorubicin in a free form and attached to carbon nanotubes as potential drug carrier, Langmuir technique has been employed. Drug in a free form was dissolved in the subphase, on which monolayers of DPPTE were formed. In case of DNR attached to carbon nanotubes, it was impossible to dissolve the carbon nanotube adducts in the subphase due to their insufficient solubility in water. Therefore, the mixed layers composed of DPPTE and SWCNTs-DNR adduct were prepared with either prevailing weight ratio of nanotubes or prevailing weight ratio of thiolipid. The isotherms are shown in Figure 2.

**Figure 3:**
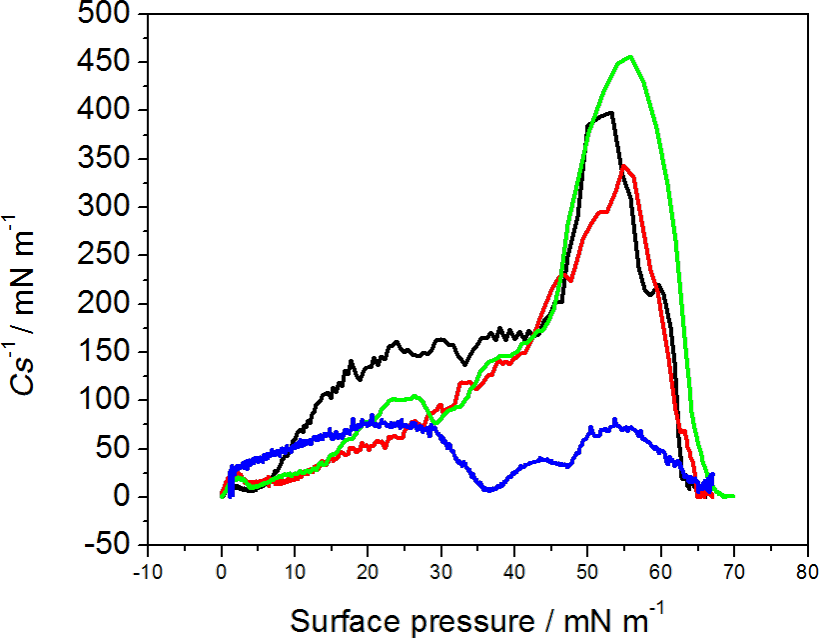
Compression modulus versus surface pressure plot for DPPTE monolayer on water subphase (black), mixed layers of DPPTE/SWCNTs-DNR weight ratio of 1:5 (red), mixed layers of DPPTE/SWCNTs-DNR weight ratio of 10:1 (green), DPPTE monolayer on subphase containing 10^−5^ DNR (blue).

Despite the fact that the experimental procedure differed in case of the two investigated forms of the drug (free drug was dissolved in the subphase, while drug-carbon nanotube adduct was mixed with thiolipid and administered as a mixture at the air–water interface), which makes the direct comparison more difficult, some interesting and new insight into such interactions may be still provided. It can be clearly observed that in the presence of daunorubicin in a free form in the subphase the shape of the isotherm changes significantly and the isotherm is shifted towards larger areas per molecule (Figure 2). As observed in our previous studies concerning the influence of DNR on thiolipid model membranes [24], characteristic parameters describing the properties of the monolayer are also significantly changed (Table 1). The area per molecule in a well-organized monolayer increases from the value of 44 Å^2^ corresponding to the DPPTE monolayer formed on pure water subphase to the value of 74 Å^2^ in the presence of the drug in the subphase. Such a significant increase is caused by the incorporation of DNR molecules into the thiolipid layer. Additionally, the shape of the isotherms is altered and the broad plateau region corresponding to the phase transition is clearly visible at relatively high surface pressure of 38 mN/m (Figure 2). Significant changes concern the compression modulus and its maximum value. The compression modulus is defined as [26]:

[1]
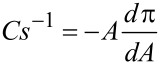


where *A* is area per molecule, and π is surface pressure. Compression modulus gives information on the state, in which the monolayer is at a given surface pressure. The maximum value of compression modulus obtained for DPPTE monolayers formed on pure water subphase is equal to 395 mN/m, which corresponds to the solid state of the monolayer [27]. In the presence of DNR in the subphase the maximum value decreases to 80 mN/m (Table 1), which indicates liquid expanded phase of the monolayer (Figure 3).

**Table 1:** Characteristic parameters of DPPTE Langmuir monolayers in the presence of free daunorubicin and daunorubicin attached to carbon nanotubes.

Substance	*A*_0_/Å^2^	*A*_coll_/Å^2^	*π*_coll_/mN m^−1^	*Cs*^−1^/mN m^−1^

DPPTE	44.5 ± 0.3	36.6 ± 0.2	65.2 ± 1.7	395 ± 25
DPPTE + 10^−5^ DNR	73.8 ± 2.6	33.5 ± 2.6	64.4 ± 2.0	80 ± 5
DPPTE/SWCNTs-DNR 1:5	50.4 ± 0.4	39.2 ± 0.3	64.7 ± 0.8	325 ± 15
DPPTE/SWCNTs-DNR 10:1	48.6 ± 0.7	39.0 ± 0.1	63.8 ± 0.3	450 ± 10

Different situation occurs when daunorubicin attached to carbon nanotubes is introduced into DPPTE monolayers. In case of mixed DPPTE–SWCNTs-DNR layer the isotherms are only slightly altered. When the amount of drug carrier is relatively small compared to the prevailing amount of the lipid (DPPTE/SWCNTs-DNR weight ratio of 10:1), the isotherm shape is not significantly changed (Figure 2). The area per molecule increases by several angstroms from the value of 44 Å^2^ corresponding to the DPPTE monolayer formed on pure water subphase to the value of 49 Å^2^, but other characteristic parameters such as collapse area and collapse pressure do not change greatly (Table 1). Interestingly, the presence of small amount of SWCNTs leads to the increase in the maximum value of compression modulus compared to DPPTE monolayers formed on water subphase (Figure 3). However, the presence of bigger amounts of carbon nanotubes in the mixed layers (DPPTE/SWCNTs-DNR weight ratio of 1:5) affect the DPPTE monolayer more significantly. Although the increasing amount of carbon nanotubes in the mixed layer does not lead to a significant increase in the area per molecule compared to the value obtained for mixed DPPTE–SWCNTs-DNR monolayers with the prevailing amount of the lipid (Table 1), more pronounced changes are observed in case of the compression modulus. Its value decreases to 325 mN/m, which is 70 mN/m less than the value reported for pure DPPTE monolayer, but it is still in the range corresponding to the solid phase. It should be also noted that for the same weight ratio of the components there is no significant difference between the effect of the unmodified CNTs and CNTs–drug adducts in the mixed layer. Since the molar percentage of the DNR in the adduct is equal only to 0.15% (see Experimental section), the absence or presence of the drug on the nanotubes will not result in the detectable changes in the isotherms, as has been shown in control experiments. The isotherms obtained for mixed layers containing DPPTE and carbon nanotubes with and without drug are almost identical. The exemplary DPPTE isotherms recorded in the presence of nanotubes without and following modification with the drug are shown in Supporting Information File 1, Figure S1.

The observed changes in the DPPTE monolayer behavior in the presence of both free drug and drug attached to carbon nanotubes should be explained in terms of the different driving forces responsible for the interactions. In case of free daunorubicin interactions, such significant changes in the isotherm shape and dramatic decrease in the maximum value of compression modulus implies that upon the incorporation into DPPTE monolayer, DNR disorganizes the thiolipid monolayer and causes its fluidization. Changes in the properties of DPPTE membrane were attributed to the electrostatic interactions [24]. Since the thiolipid molecules are negatively charged and pKa of daunorubicin is equal to 8.4 [28], at the pH corresponding to pure water there is electrostatic attraction between the negatively charged polar heads of the lipid and positively charged drug. Obviously, hydrophobic interactions between the hydrophobic part of the drug and acyl chains of the lipids should be also taken into account, but electrostatic interactions seem to be playing the most important role, especially in case of the interactions at lower surface pressures, when the drug dissolved in the subphase first interacts with polar heads of the lipid. However, with the increasing surface pressure DNR molecules may penetrate deeper into the monolayer and then hydrophobic interactions also play important role. Consequently, those types of interactions result both in the observed changes in the *Cs*^−1^ values, as well as in the significant increase in the area per molecule in the organized layer (Table 1) as reported before [24].

In case of the effect of SWCNTs modified with DNR different explanation may be given. The unexpected increase in the maximum value of compression modulus for the mixed layers with the prevailing amount of thiolipid may be caused by the fact that due to the presence of relatively small amounts of carbon nanotubes in the monolayer the thiolipid molecules become more oriented with less tilted hydrocarbon chains, which leads to the observed increase in the maximum value of compression modulus. In the same time, the nanotubes occupy the interfacial area and therefore a small increase in the area per molecule is observed (Table 1). The increasing amount of the nanotubes in the mixed layer results in some fluidization of the DPPTE monolayer because the increasing number of nanotubes prevents from the hydrophobic interactions between acyl chains of the lipid and thus leads to the disorganization of the layer. However, this influence is not as significant as the influence of DNR in the free form and therefore it does not introduce the change of the phase of the monolayer. Additionally, the relatively small increase in the area per molecule with the increasing content of the nanotubes in the thiolipid monolayer (from 48.6 to 50.4 Å^2^ for the prevailing weight ratio of the lipid and the nanotubes, respectively, Table 1) indicates that the nanotubes may form aggregates or bundles and do not distribute evenly within the thiolipid monolayer. They may be also squeezed out from the monolayer into the subphase or into the close vicinity of the monolayer. Alternatively, closing the barriers and increasing the surface pressure may lead to the above mentioned formation of aggregates or bundles. Moreover, in the presence of SWCNTs-DNR conjugates the isotherms are only slightly shifted towards larger areas per molecule but their shape is not altered. Similar observation was made by Cancino et al., who studied the effect of carbon nanotubes-dendrimers nanoconjugates on DPPC monolayers [29]. The presence of such types of adducts did not significantly affect the properties of lipid monolayers.

In conclusion, the effect of the free drug and drug attached to the potential carrier on the properties of model thiolipid monolayer is different. Daunorubicin in the free form significantly changes the properties of DPPTE monolayers by increasing the area per molecule in the monolayer and causing their immense fluidization. The electrostatic interactions between positively charged drug and negatively charged polar headgroups of the lipid are most relevant in this case. The influence of carbon nanotubes modified with DNR on the properties of DPPTE monolayers is less pronounced. The observed areas per molecule are only slightly increased compared to pure DPPTE monolayer, which implies that although CNTs occupy the interfacial area, the change in the organization of the lipid molecules is not as big as in case of a free drug. It may be explained by a different mechanism responsible for the interactions in this case. Although the amine group of DNR attached to the ends of carbon nanotubes is protonated as in a free drug, which still gives the possibility of the electrostatic interactions, the hydrophobic interactions with nanotubes seem to play the crucial role. First of all, the drug modification of the nanotubes provides relatively small mole fraction of the drug with respect to the nanotube, as stated in the Experimental section. Secondly, the modification at the end (and defect sites) of the nanotubes leaves the sides of the nanotubes unmodified. Therefore, due to the strong hydrophobicity of the nanotubes one may suppose that although electrostatic interactions between the attached drug and polar heads of the lipid are still possible, the hydrophobic interactions between carbon nanotubes and acyl chains of the lipids will be determining the overall interactions in this case.

### Electrochemical studies

**Scheme 1:**
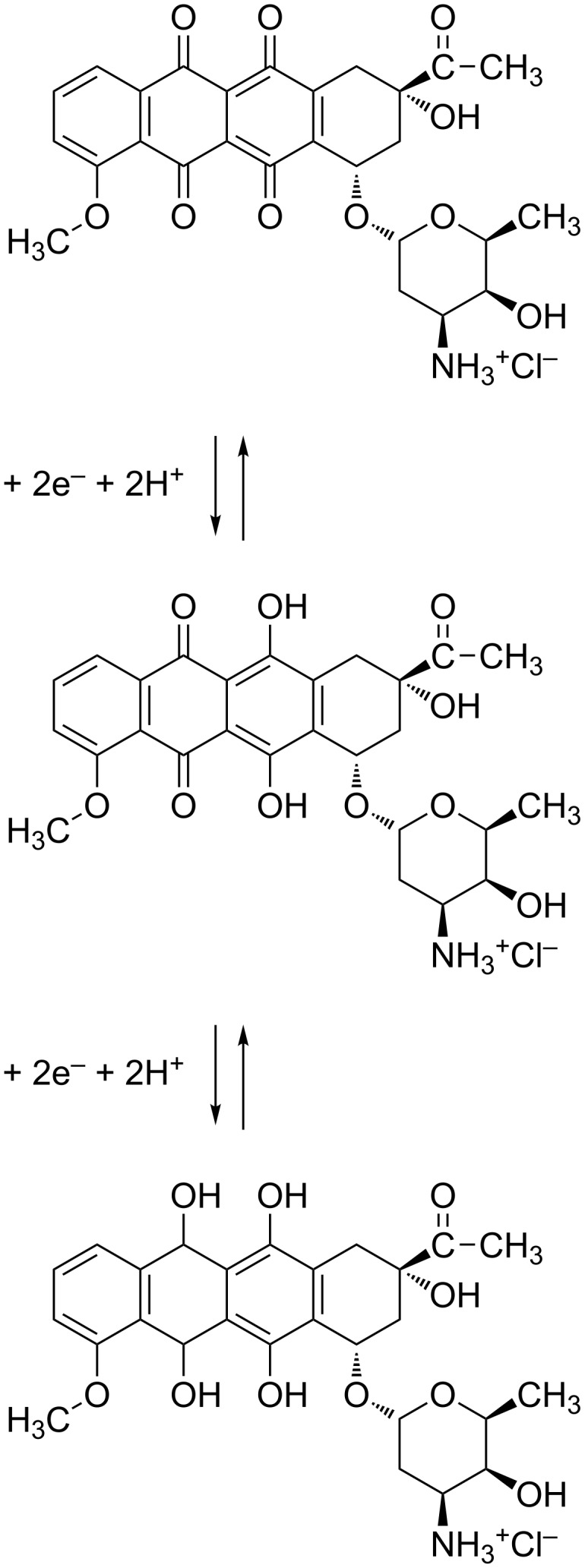
The electrode processes of daunorubicin.

**Figure 4:**
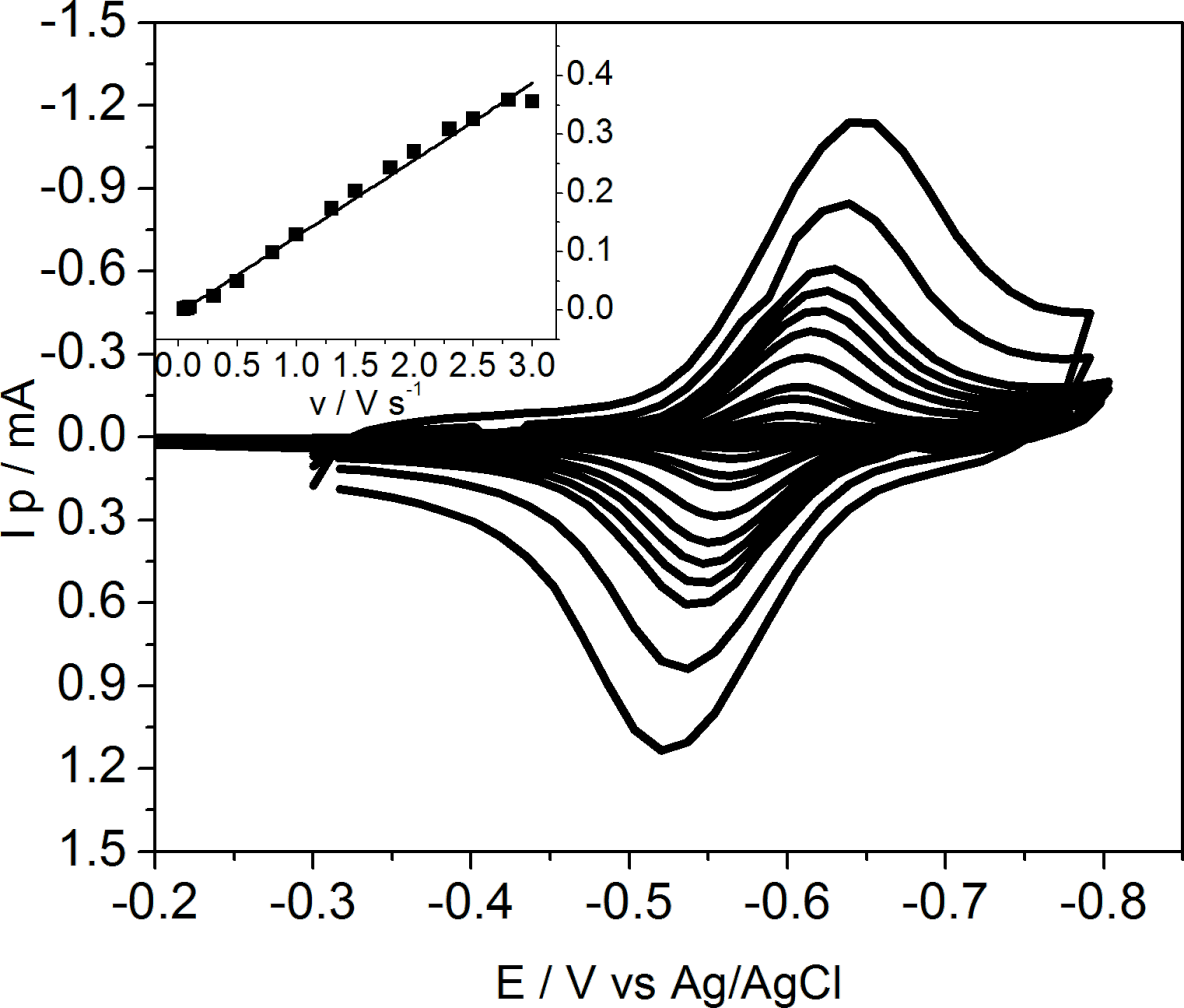
Cyclic voltammograms recorded at the increasing scan rate for mixed DPPTE/SWCNTs-DNR (1:5 w/w) layers transferred onto gold electrodes by means of LB method for end modification of nanotubes. Inset: dependence of peak current vs scan rate.

Since daunorubicin is electroactive and undergoes 2e^−^/2H^+^ redox process (Scheme 1), the interactions of the drug in a free form and attached to carbon nanotubes with model membranes may be also followed using electrochemistry. After the LB transfer described in detail in Experimental section, supported layers were characterized by electrochemical techniques. First, in order to verify the presence of daunorubicin attached to carbon nanotubes in the mixed layer on the electrode, cyclic voltammetry was performed. The reduction–oxidation peaks observed in voltammograms correspond to the electrode process of the quinone-hydroquinone group (Figure 4) [30]. Basing on the results obtained for the supported mixed DPPTE–SWCNTs-DNR monolayers with the prevailing amount of nanotubes it has been shown that the drug is present at the electrode surface. Additionally, the linear dependence of the peak current versus the scan rate proves the adsorptive character of the observed electrode process and thus confirms that the electroactive drug is covalently attached to the carbon nanotubes supported at the electrode surface.

In order to assess the interactions of the free daunorubicin with model membranes, DPPTE monolayers were transferred by means of LB method onto gold electrodes and were exposed to 10^−5^ M DNR solutions for 600 s to ensure the incorporation of the drug into the model membrane. The optimal incubation time of the model membrane in the drug solution was based on our previous studies, which showed that after this time cyclic voltammograms become stable [24]. Alternatively, it is also possible to incorporate the drug into the model membrane during its formation and then transfer it onto the electrode [31]. It should be also stressed that the concentration used for the incubation of the modified electrodes is consistent with the DNR concentration used in monolayer studies. Additionally, it corresponds to the concentrations used in the in vitro studies. The IC_50_ value, which is defined as the concentration of a drug that inhibits cell growth by 50%, given in the literature usually varies from 10^−6^ M to 10^−5^ M depending on the type of cell lines [32–34].

**Figure 5:**
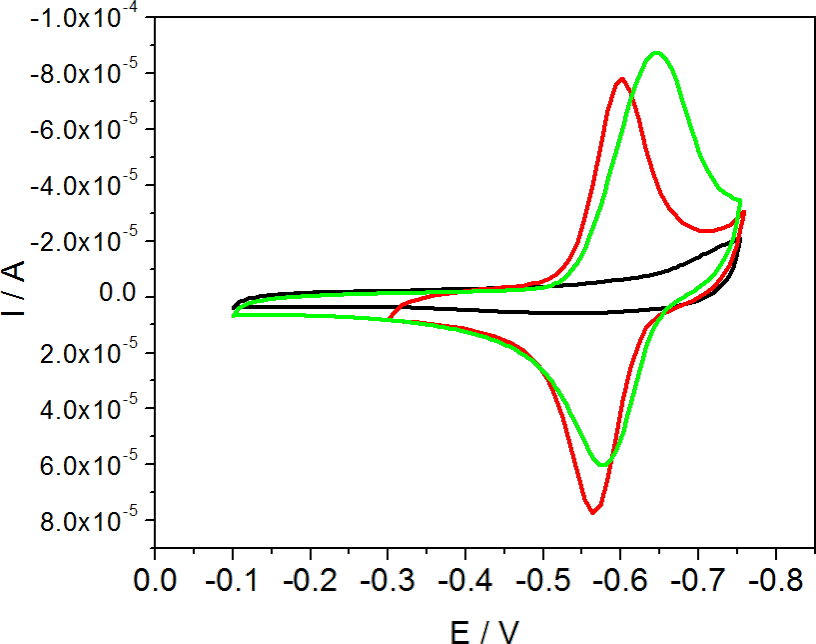
Cyclic voltammograms recorded for gold electrode modified with DPPTE monolayer (black), mixed DPPTE/SWCNTs-DNR (1:5 w/w) layer (red) and DPPTE monolayer exposed to 10^−5^ M DNR solution for 600 s (green). Scan rate: 500 mV/s.

In the next step, voltammograms obtained after the incubation in DNR solution were compared with the voltammograms recorded for electrodes modified with DPPTE monolayer (before exposure to DNR) and electrodes modified with mixed DPPTE–SWCNTs-DNR monolayers (Figure 5). In case of both DPPTE monolayers exposed to DNR solution and mixed DPPTE–SWCNTs-DNR monolayers the oxidation–reduction peaks corresponding to the electrode process of daunorubicin are observed and the peak currents are similar. The control experiments on the effect of unmodified SWCNTs in the supported layers are not shown because in the absence of electroactive species such as daunorubicin attached to carbon nanotubes on the electrode surface, no reduction–oxidation peaks can be observed, since neither unmodified carbon nanotubes, nor thiolipid undergo reduction–oxidation processes in the potential range investigated. Therefore, in such case only the blocking of the electrode by the mixed DPPTE–SWCNTs-bare could be seen, similarly to the blocking of the electrode by the monocomponent DPPTE monolayer (Figure 5).

In order to compare the results for the two types of modified electrodes in a more detailed way, surface concentration of the drug may be calculated. The surface concentration of daunorubicin can be estimated using the following equation:

[2]
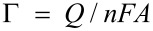


where Γ is surface concentration (mol/cm^2^), *Q* is charge under cathodic peak (C), *n* is number of electrons, *F* is Faraday constant and *A* is electrode area (cm^2^). The surface concentration calculated based on the cyclic voltammogram for the electrode modified with mixed DPPTE–SWCNTs-DNR monolayer is equal to 0.67 × 10^−10^ mol/cm^2^, while the value obtained for DPPTE supported monolayer exposed to DNR solution is equal to 0.60 × 10^−10^ mol/cm^2^ as reported before [24]. Moreover, the values obtained for the mixed layer containing modified carbon nanotubes are comparable to the values reported for carbon nanotubes derivatized with antraquinone [35] and other types of electroactive species [36–37].

It is interesting to compare the surface concentrations of the drug incorporated into the supported model membranes in the two forms: free and attached to drug carriers. However, due to different solubilities, the experimental procedures are different: free drug is more soluble and, therefore, can be dissolved in the subphase from which it penetrates the lipid monolayer, while drug–carbon nanotube adduct is added to the thiolipid and chloroform and next the mixed sample is placed at the air–water interface. Therefore, the initial concentrations of the drugs cannot be easily compared. Our solution was to transfer the layer onto a solid substrate (electrode) and evaluate the amount of the drug present in the model membrane supported on the electrode by electrochemistry. In case of daunorubicin in the free form the estimation was based on the DNR concentration in the subphase (10^−5^ M) and surface concentration of the drug incorporating into the model membrane precompressed to 30 mN/m [24], while in case of DNR attached to carbon nanotubes the content of DNR (1.25 × 10^−7^ mol of drug per 1 mg of carbon nanotubes) in the total amount of the DPPTE–SWCNTs-DNR adduct (with respect to the DPPTE/SWCNTs-DNR ratio) administered at the air–water interface as a mixed layer and transferred onto the electrode was taken into account. The estimated amount of daunorubicin in the supported model membrane is equal to 2.4 × 10^−13^ and 2.1 × 10^−13^ mol for the free drug and drug attached to carbon nanotubes, respectively. It should be noted that the surface concentration of DNR calculated based on electrochemical experiments is also the same for both mixed DPPTE–SWCNTs-DNR monolayer and incorporated drug in the free form. This proves that the amount of the drug in the model membrane is similar for both types of the drug delivery: direct or bound to the nanocarrier. However, basing on the results of monolayer studies, the effect of SWCNTs-DNR adducts on the properties of model DPPTE membranes is much smaller than the one observed for a free drug (Figure 2 and Figure 3). It is especially important in the view of the application of carbon nanotubes as drug carriers. These results prove that CNTs modified with DNR may provide the same amount of the therapeutic agent as the drug in the free form but in the same time they do not influence the organization and properties of the membranes to such extent as the free drug.

## Conclusion

Interactions of anticancer drug daunorubicin with model thiolipid membranes were investigated using Langmuir technique and electrochemical methods. The drug was either in the free form dissolved in the subphase, on which the DPPTE monolayer was formed, or attached to single-walled carbon nanotubes, which are potential drug carriers. In this case, drug–carrier adducts were deposited onto the air–water interface forming the mixed layers of DPPTE with differing weight ratio of the components. Langmuir studies revealed that DNR influences the properties of the thiolipid monolayer leading to a significant increase in the area per molecule. Additionally, the organization of the monolayer changes: the maximum value of the compression modulus implies that upon incorporation of the drug in the free form, the layer changes its phase from solid to liquid. The observed differences in the properties of the monolayer are caused by the electrostatic interactions between the positively charged drugs and negatively charged polar heads of the lipid. Results of the studies with SWCNTs-DNR conjugates revealed that their influence on DPPTE monolayers is much less pronounced. The characteristic parameters such as area per molecule do not change as significantly as in case of a free drug. Moreover, the organization of the monolayer is not influenced to such a large extent and even at the higher weigh content of CNTs in the mixed layer, the monolayer retains its solid character. In this case hydrophobic interactions between the nanotubes and DPPTE molecules seem to play the most important role.

Electrochemistry was also employed to compare the model membranes containing daunorubicin in the two investigated forms. Cyclic voltammetry performed for the electrodes modified with DPPTE monolayers containing daunorubicin either in the free form or attached to carbon nanotubes proved the presence of the electroactive drug at the electrode surface. Moreover, it was shown that the surface concentration of the drug on the electrode surface is similar for both the drug in a free form and the drug–nanocarrier adduct. This observation proves that application of single-walled carbon nanotubes as drug delivery system allows for the transport of the comparable amount of the drug with respect to the drug in a free form but the influence of the drug–carrier adduct on the model membranes is much smaller. This conclusion is important from the point of view of side effects of the drug treatment and confirms the efficiency of the application of carbon nanotubes as drug delivery systems.

## Supporting Information

File 1 DPPTE isotherms.
