# Supplementary material for: Comparison of the interactions of daunorubicin in a free form and attached to single-walled carbon nanotubes with model lipid membranes
Source: Beilstein J Nanotechnol. 2016 Apr 8;7:524–32. doi: 10.3762/bjnano.7.46 (PMC4901540; doi:10.3762/bjnano.7.46)
Supplement: File 1 — DPPTE isotherms. [file Beilstein_J_Nanotechnol-07-524-s001.pdf]

Supporting Information

for

Comparison of the interactions of daunorubicin in a free form and attached to single-walled carbon nanotubes with model lipid membranes

Dorota Matyszewska

Address: Faculty of Chemistry, Biological and Chemical Research Centre, University of Warsaw, Żwirki i Wigury 101, 02089 Warsaw, Poland

Email: Dorota Matyszewska - dorota.matyszewska@chem.uw.edu.pl

DPPTE isotherms

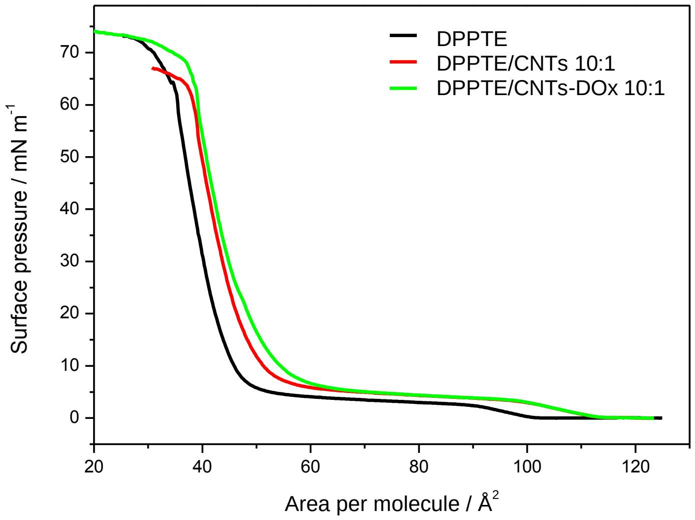

Figure S1: Surface pressure-area per molecule (π–A) isotherms DPPTE monolayer on water subphase (black), mixed layers of DPPTE/SWCNTs-PEG weight ratio of 10:1 (red), mixed layers of DPPTE/SWCNTs-PEG-DOx weight ratio of 10:1 (green).

Isotherms obtained for pure DPPTE monolayer and mixed monolayers of DPPTE and carbon nanotubes modified with functional groups and PEG prior to the addition of anticancer drug (doxorubicin) and with the drug attached to the nanotubes.
